# Efficient Synthesis of Glutamate Peptide-Estradiol Conjugate for Imaging Estrogen Receptor-Positive Diseases

**DOI:** 10.1155/2018/5208964

**Published:** 2018-09-25

**Authors:** Ming-Chi Shih, Sergio Daniel Simon, Zhiming Jin, Yuan Gui, Bohua Xu, Zhihong Xu, Paulo Henrique Rosado-de-Castro, Ana Maria Silveira Braghirolli, Lea Mirian Barbosa da Fonseca, Tomio Inoue, David J. Yang

**Affiliations:** ^1^Department of Internal Medicine, School of Medicine, Federal University of Rio De Janeiro, Rio De Janeiro, Brazil; ^2^Brazilian Breast Cancer Study Group (GBECAM), Brazil; ^3^Jiangsu Huayi Technology Co., Ltd, Jiangsu, China; ^4^Institute of Biomedical Sciences, Federal University of Rio de Janeiro, Rio de Janeiro, Brazil; ^5^Department of Radiology, School of Medicine, Federal University of Rio de Janeiro, Rio de Janeiro, Brazil; ^6^Nuclear Engineering Institute, National Nuclear Energy Commission, Rio de Janeiro, Brazil; ^7^Director of Advanced Medical Center, Shonan Kamakura General Hospital, Kamakura, Japan; ^8^Vyripharm Biopharmaceuticals LLC, Houston, Texas, USA

## Abstract

Molecular imaging of estrogen receptor-positive (ER+) pathway-activated system serves the basis of ER+ disease management such as cancers and endometriosis. ER+ patients have better response to endocrine therapy and survive twice as long as negative ER patients. However, tumor resistance resulting from clinical used aromatase inhibitors and antiestrogens is unpredictable. Radiolabeled ER+ ligand could quantify ER+ tissue uptake which helps to stage and restage of the cancer as well as endometriosis. The differential diagnosis of ER+ lesions by using a labeled ligand helps to select the patients for optimal response to endocrine therapy and to discontinue the treatment when resistance occurs. In addition, radiolabeled ER+ ligand serves as basis for image-guided response follow-up. Glutamate receptors are cell surface receptors which are overexpressed in inflammation and infection. Using glutamate peptide as a drug carrier helps to target intracellular genes via glutamate receptor-mediated process. Reports have shown that polyglutamate is a drug carrier that could alter drug solubility and enhance estrogen receptor-ligand binding pocket. However, polyglutamate was a blend of mixed polymer with a wide range of molecular weight. Thus, the structural confirmation and purity of the conjugates were not optimized. To overcome this problem, the efficient synthesis of glutamate peptide-estradiol (GAP-EDL) conjugate was achieved with high purity. EDL was conjugated site-specific at the first glutamate of GAP. The average cell uptake of ^68^Ga-GAP-EDL was 5-fold higher than the previous reported synthesis. The efficient synthesis of GAP-EDL has greatly enhanced sensitivity and specificity in cell uptake studies. In vivo PET imaging studies indicated that ^68^Ga-GAP-EDL could image ER (+) tumors in MCF-7 tumor-bearing mice. Therefore, GAP-EDL makes it possible to image ER-enriched endometriosis and cancer.

## 1. Introduction

Estrogen leads to genomic effects on transcription through *α* and *β* estrogen receptors (ERs), which are primarily found in the nucleus. The function of ER*α* in the mediation of gene transcription is widely documented, and reports with mouse models and human breast cancer cell lines have indicated that ER*α* has a part in cell proliferation. On the other hand, the function of ER*β* as a transcriptional regulator continues to be ambiguous. Reports indicate that ER*β* can reduce ER*α* activity, possibly by heterodimerization [1, 2]. Estrogen may elicit rapid ‘non-genomic' actions on different cellular processes through membrane ERs. ER modulators including tamoxifen are valuable instruments for the evaluation the mechanisms of action of estrogen. ER intermediates inhibition of NF-*κ*B activity at numerous levels. This cross-talk among central regulators of endocrine and immune systems might be used for treating oncologic, inflammatory, and autoimmune disorders [3]. Oxidative stress is related to an excess of reactive oxygen species (ROS). ROS induce DNA injury and lead to overactivation of poly-ADP ribose polymerase-1 (PARP-1), which results in depletion of cytosolic NAD+. ROS also increase proinflammatory transcription factor NF-kB leading to neuroinflammation. Reduction of NAD+ inhibits glycolysis and yielding inadequate levels of pyruvate and reducing mitochondrial ATP production. Furthermore, NAD+ reduction causes ineffective conversion of lactate to pyruvate and lactate cannot be the energy substrate, leading to cell apoptosis. There are three principal paths for the elimination of ROS, including reduced thioredoxin (TXN), glutathione (GSH), and catalase, which have key enzymes that may be aimed to reduce antioxidant effects within cancer cells. Amid such pathways, GSH is influenced by increased cysteine and glutamate transporter systems, as well as NADPH. GSH is derived from cysteine and glutamate. Glutaminase 1 (GLS1) and GLS2 produce glutamate, and cysteine is provided by the cystine/glutamate transporter XCT for the production of GSH through the action of the glutamate–cysteine ligase modifier subunit (GCLM) and the GCL catalytic subunit (GCLC). GSH has direct action on the elimination of ROS through glutathione peroxidase and glutathione* S*-transferase [4–6]. Glutamate leads to cellular overload with Ca2+ ions during ROS which induces the production of GSH. Thus, glutamate receptor/transporter system is associated with intracellular GSH production. The excitatory amino acid glutamate exerts its action through different glutamate receptors. Metabotropic glutamate receptors may interact with membrane ERs and, more specifically, the mGlu5 receptor subtype. mGlu5 and 17*β*-estradiol antagonists have neuroprotective effects in the 1-methyl-4-phenyl-1,2,3,6-tetrahydropyridine (MPTP) mouse model of Parkinson disease [7]. Therefore, estradiol (EDL), an ER ligand, was selected as a molecule for conjugation to glutamate peptide.

It has been described that glutamate peptide (GAP) stimulates bone resorption in vitro and specific to glutamate receptors [8]. GAP has shown its ability to target renal tissue [9]. It would be amenable to conjugate estradiol (EDL) to GAP and GAP-EDL may increase binding efficacy to cytosolic ERs. With acid residue from GAP, GAP could chelate radiometallic isotopes for imaging and radio therapeutic applications. Previously it has been reported that ^99m^Tc-GAP-EDL and ^68^Ga-GAP-EDL are useful compounds for imaging functional ER via ER-mediated process by planar scintigraphy and PET [10, 11]. However, EDL was conjugated to GAP using aqueous purification [10–12]. However, the cell uptake was low. This is probably due to its purity. In receptor-based imaging, the agent needs to achieve high specific activity (Ci/umol). If the labeled agent is contaminated with different molecular weight, the image quality assurance would be problematic. In addition, it is unknown at which position the drug is conjugated to GAP. Regulatory compliance of detailed Chemistry, Manufacturing, and Control (CMC) information during clinic trials is required by governmental agency. Thus, it is amenable to develop pure glutamate peptide-estradiol (GAP-EDL). With sufficient purity, the overexpressed glutamate systems could enhance cell uptake of labeled GAP-EDL. Once GAP-EDL internalized, GAP-EDL could target ER pathways. Here, we report the efficient synthesis of GAP-EDL that allows EDL to be conjugated to the first glutamate of glutamate peptide.

## 2. Material and Methods

### 2.1. Synthesis of (8R,9S,13S,14S)-3-Cyanomethoxy-13-methyl-7,8,9,11,12,14,15,16-octahydro-6H-cyclopenta[a]phenanthren-17-one (GAP-EDL-1)

**Figure 1:**
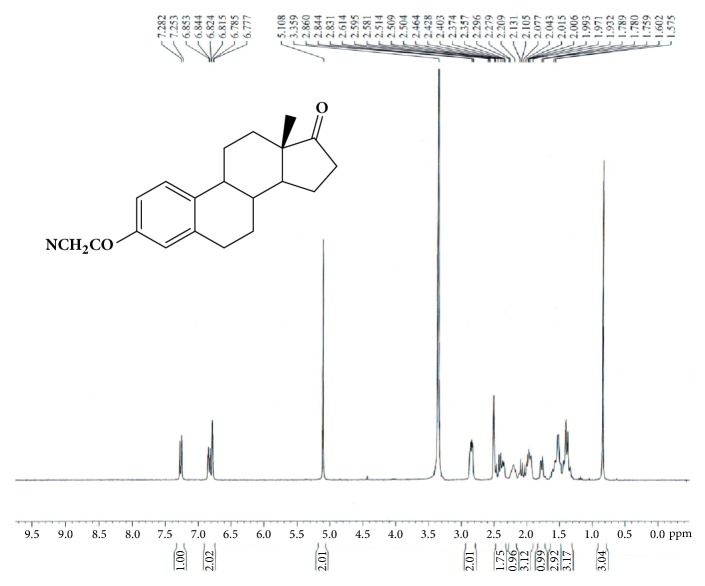
Synthesis of GAP-EDL-1.

(8R,9S,13S,14S)-3-Hydroxy-13-methyl-7,8,9,11,12,14,15,16-octahydro-6H-cyclo-penta[a] phenanthren-17-one (Estrone, 2.00g, 7.40 mmol) was dissolved in anhydrous tetrahydrofuran (THF, 25ml) under nitrogen atmosphere. Sodium methoxide (0.80g, 14.82mmol) was then added. Bromoacetonitrile (1.78g, 14.84 mmol) dissolved in 12mL THF was dropwisely added. The mixture was stirred at room temperature for 1h. The additional sodium methoxide (0.60g, 11.11mmol) and bromoacetonitrile (1.21g, 10.89 mmol) in 4mL THF were added. The reaction mixture was continued to stir at room temperature for 0.5 h. Saturated ammonium chloride solution (100ml) and ethyl acetate (100mL) was added to the reaction mixture. The organic layer was collected. The aqueous phase was extracted with additional ethyl acetate (2x50mL). The organic layer was combined and washed with saturated sodium chloride solution. The organic extract was dried over magnesium sulfate and filtered. The solvent was evaporated under reduced pressure. The crude solid product was washed with diethyl ether and obtained 2.11g (6.82mmol) solid product with 92.2% yield. ^1^H-NMR (300MHz, DMSO): 7.27 (d, J=8.6Hz, 1H), 6.83 (dd, J=8.8Hz,2.6Hz, 1H), 6.78(d, J=2.5Hz, 1H), 5.11(s, 2H), 2.80-2.86(m, 2H), 2.36-2.50(m, 2H), 2.16-2.26(m, 1H), 1.84-2.15(m, 3H),1.74-1.80(m, 1H), 1.33-1.46(m, 3H), and 1.46-1.60(m, 3H),0.84 (s, 3H). LC-MS was calculated for C20H23NO2, 309.2, and found [M+H] 310.1. The spectrum is shown in Figure 1.

### 2.2. Synthesis of (8R,9S,13S,14S,17S)-3-Aminoethoxy-13-methyl-6,7,8,9,11,12,14,15,16,17-decahydrocyclopenta[a]phenanthrene-17-ol (GAP-EDL-2)

**Figure 2:**
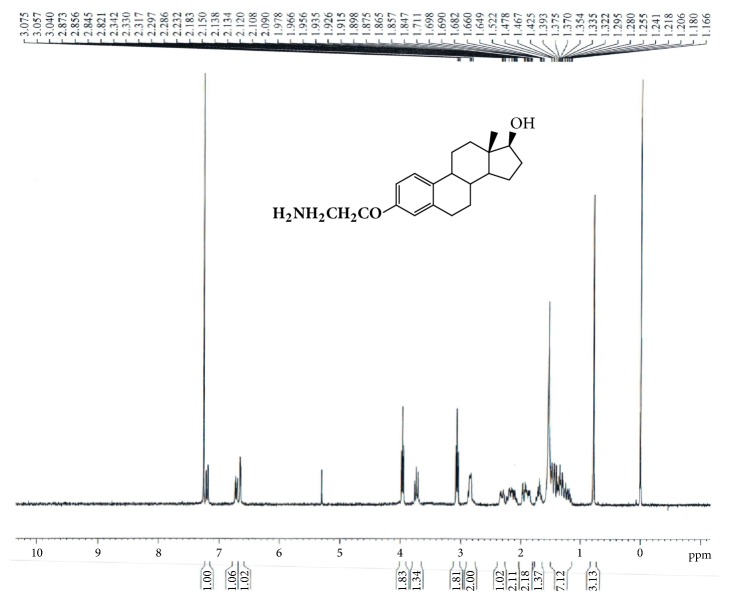
Synthesis of GAP-EDL-2.

Under nitrogen atmosphere GAP-EDL-1 (2.10g, 6.79mmol) was dissolved in anhydrous THF (90 ml). The mixture was cooled down to 0-5°C and lithium aluminum hydride (1.70g, 44.80mmol) was portion wisely added. The reaction mixture was stirred at 0-5°C for 5-10min and then at room temperature 2 hours. After reaction was ended, the mixture was cooled down to 0-5°C again and quenched with water. The suspension was filtered and washed with THF. The filtrate was evaporated and concentrated under reduced pressure. The crude compound was purified by the silica gel-packed column chromatography eluted with methanol/dichloride (MeOH/DCM; 1/30) to afford 3-aminoethyoxy estradiol (GAP-EDL-2) as off-white solid (1.22 g,3.87mmol, 57.0 % yield).^1^H-NMR (300MHz, CDCl3): 7.20(d, J=8.6Hz, 1H), 6.71 (dd, J=8.6Hz,2.7Hz, 1H), 6.64(d, J=2.7Hz, 1H), 3.98(t.J=5.2Hz,2H), 3.73(t.J=8.4Hz,1H), 3.06(t.J=5.2Hz,2H), 2.80-2.85(m, 2H), 2.26-2.36(m, 1H), 2.06-2.23(m, 2H), 1.83-1.98(m, 2H),1.65-1.76(m, 2H), 1.65-1.76(m, 1H), and 1.14-1.48(m, 7H),0.78 (s, 3H). LC-MS was calculated for C20H29NO2, 315.2, and found [M+H] 316.1. The spectrum is shown in Figure 2.

### 2.3. Synthesis of 5- N- [Amino-3-ethoxyestradiol]-1-Benzyl-N-tert-butoxycarbonyl-L-Glutamate 5-Amide (GAP-EDL-3)

**Figure 3:**
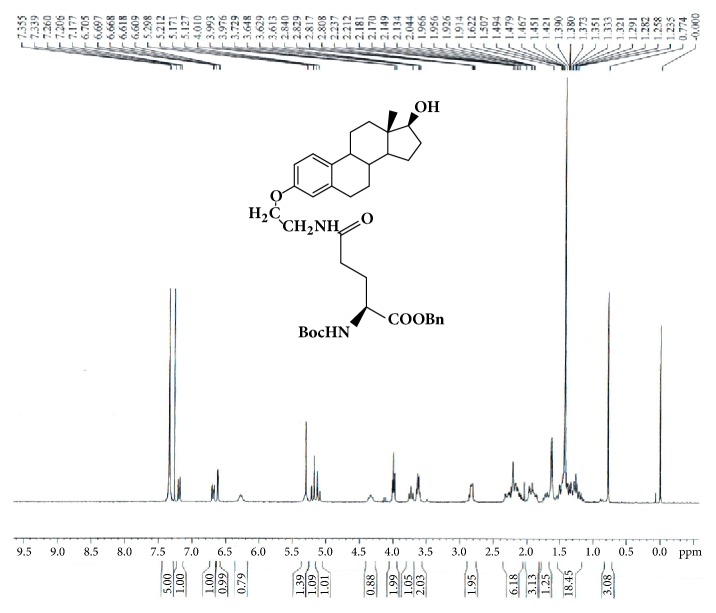
Synthesis of GAP-EDL-3.

1-Benzyl-N-tert-butoxycarbony-L-glutamic acid ester (0.50g, 1.48mmol), 1,2,3-benzotriazol-1-ol (0.215g,1.59mmoml) and benzotriazol-1-yloxy-tris(dimethylamino)phosphonium hexafluoro-phosphate (BOP, 0.700g,1.58mmol) were dissolved in anhydrous dimethylformamide (DMF, 15 mL) under nitrogen atmosphere. N,N-Diisopropylethylamine (DIPEA, 0.205g,1.59mmol) was then added. The reaction mixture was stirred at 0-5°C and then added with GAP-EDL-2 (0.50g, 1.585mmol). The reaction was stirred at 0-5°C for 10min and at room temperature for 1 hour. After completion of reaction, DMF was removed under reduced pressure and ethyl acetate (250 ml) was added. The organic phase was washed with 4% sodium bicarbonate solution (200mL), dried over magnesium sulfate, filtered, and concentrated under reduced pressure. The crude compound was purified by a column chromatography over silica gel eluted with ethyl acetate/dichloromethane (EtOAc/DCM; 5/1) to afford GAP-EDL-3 as white solid (0.40g, 0.63mmol, 42.5% yield). ^1^H-NMR (300MHz, CDCl3):7.31-7.38(m,5H), 7.19 (d, J=8.7Hz, 1H), 6.69 (dd, J=8.6Hz, 2.7Hz, 1H), 6.61(d, J=2.6Hz, 1H), 6.27 (br, 1H), 5.27-5.34 (m,1H), 5.19 (d, J=12.2Hz,1H), 5.11(d, J=12.2Hz,1H),4.28-4.47(m,1H), 3.99 (t, J=5.1Hz,2H), 3.73 (t, J=8.5Hz,1H), 3.62 (q, J=5.7Hz,2H), 2.78-2.88 (m, 2H), 2.06-2.34 (m, 6H),1.84-1.98 (m, 3H),1.65-1.76(m, 1H), 1.14-1.54(m, 16H), and 0.77(s, 3H). LC-MS was calculated for C37H50N2O7, 634.4, and found [M+H] 335.2. The spectrum is shown in Figure 3.

### 2.4. Synthesis of 5- N- [Amino-3-ethoxyestradiol]- N-tert-butoxycarbonyl-L-Glutamic acid 5-Amide (GAP-EDL-4)

**Figure 4:**
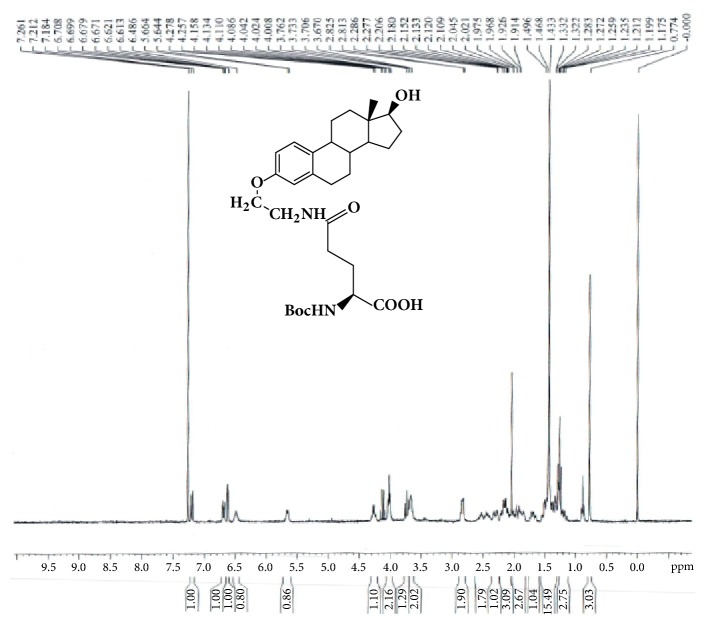
Synthesis of GAP-EDL-4.

To a solution of GAP-EDL-3 (0.39g, 0.61mmmol) in THF (40mL) and methanol (8mL) under nitrogen atmosphere, 0.080g of 5% Pd/C was added. The reaction mixture was stirred at room temperature under hydrogen atmosphere for 1 hour. The suspension was then filtered over Celite and concentrated under reduced pressure. The de-ester product was obtained as white solid (0.30g, 0.55mmol, 89.7% yield). ^1^H-NMR (300MHz,CDCl3): 7.19 (d, J=8.7Hz,1H),6.69 (dd, J=8.6Hz,2.7Hz, 1H), 6.61 (d, J=2.6Hz, 1H), 6.49 (br,1H), 5.64-5.66 (m,1H), 4.26 (q, J=6.6Hz1H), 4.02 (t, J=5.1Hz, 2H), 3.73 (t, J=8.4Hz,1H), 3.62-3.70 (m, 2H), 2.81-2.86 (m,2H), 2.38-2.60 (m,2H), 2.26-2.36 (m,1H), 2.06-2.22 (m,3H),1.82-2.04 (m,3H),1.64-1.76 (m, 1H), 1.17-1.55 (m, 16H), 0.77 (s, 3H). LC-MS was calculated for C30H44N2O7, 544.3, and found [M+H] 545.2. The spectrum is shown in Figure 4.

### 2.5. Synthesis of 5- N-[Amino-3-ethoxyestradiol]-N-tert-butoxycarbonyl-Glutamoyl-1,5-di-t-butyl-L-Glutamate pentapeptide ester (GAP-EDL-5)

**Figure 5:**
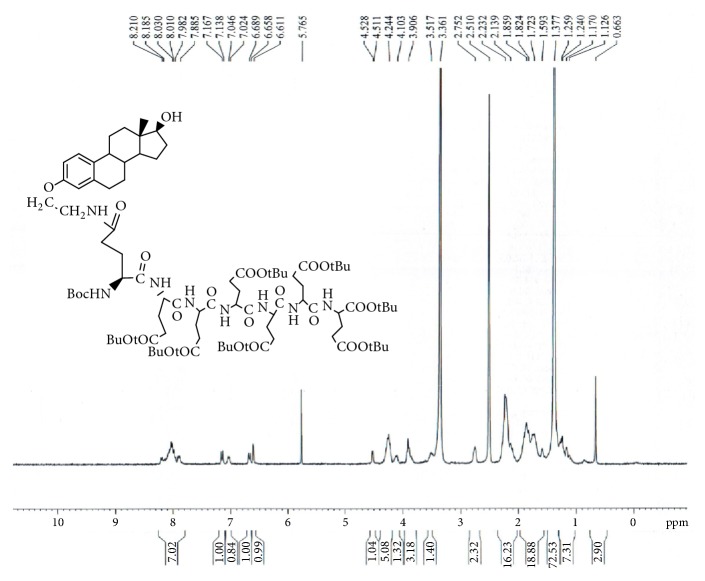
Synthesis of GAP-EDL-5.

**Figure 6:**
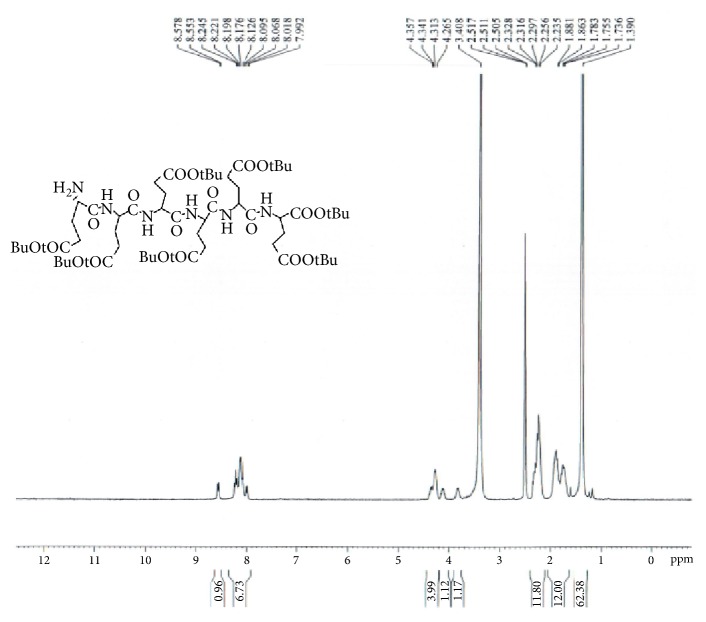
^1^H-NMR of 1,5-di-tert-butyl GAP ester.

**Figure 7:**
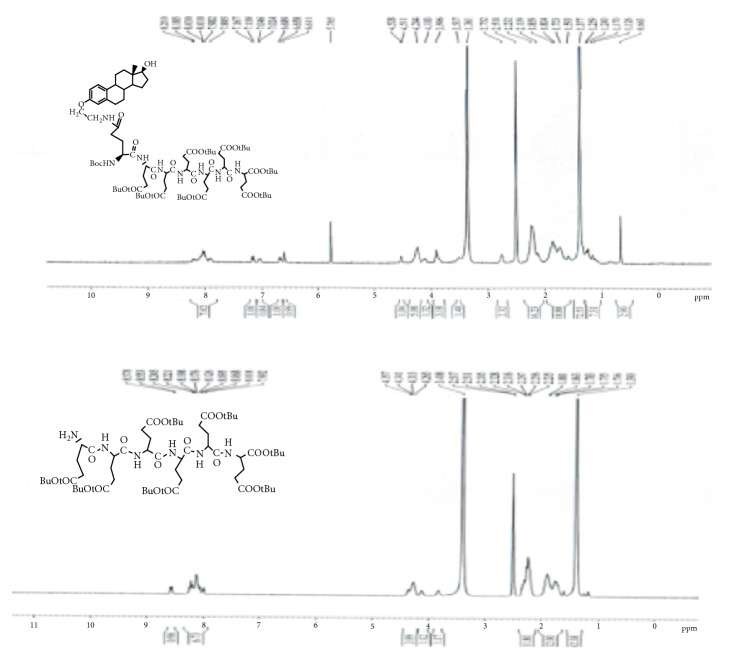
^1^H-NMR of 1,5-di-t-butyl GAP ester and GAP-EDL-5.

GAP-EDL-4 (0.580g, 1.06mmmol), 1-Hydroxybenzotriazole (0.146g, 1.08mmoml), and BOP (0.470g, 1.06mmol) were dissolved in anhydrous DMF (10 mL). The mixture was stirred under nitrogen atmosphere with temperature cooled down to 0°C. N,N-Diisopropylethylamine (DIPEA, 0.140g, 1.08mmol) and 1,5-di-t-butyl-L-Glutamate pentapeptide ester (GAP ester, 1.260g, 1.06mmmol) (Zhejiang Ontores Biotechnologies Co., Ltd, Hangzhou, Zhejiang, China) were then added. The reaction mixture was continued to stir for 16-18h at 50°C and monitored by TLC. When TLC analysis showed the reaction completion, DMF was removed under reduced pressure and DCM (250ml) was added. The organic phase was washed with saturated sodium chloride solution (200mL), dried over magnesium sulfate, filtered, and concentrated under reduced pressure. The crude compound was purified by a silica gel-packed column eluted with gradient DCM and DCM/MeOH (100:0-100:1) to afford GAP-EDL-5 as foam white solid (0.96g, 0.56mmol, 52.8% yield. ^1^H-NMR(300MHz, DMSO+D2O):7.80-8.20(m, 7H),7.12 (d, J=8.6Hz, 1H), 7.03 (brs, 1H), 6.64 (d, J=8.6Hz,1H), 6.58(s,1H), 4.04-4.28 (m,6H), 3.77-3.93(m, 3H), 3.45-3.54(m,1H), 3.30-3.44 (m, 2H), 2.66-2.78 (m, 2H), 2.00-2.32(m, 16H), 1.50-2.00(m,18H), 1.35(s, 72H), 1.05-1.28(m, 7H), and 0.63(s, 3H). The spectrum is shown in Figure 5. To ascertain the structure of GAP-EDL-5, the proton NMR of 1,5-di-t-butyl-L-Glutamate pentapeptide ester (GAP ester) was performed (shown in Figure 6). The proton NMR of GAP-EDL-5 was compared to GAP ester (Figure 7). There were chemical shifts at 7ppm seen in GAP-EDL-5, but not in GAP ester. The chemical shift was the aromatic protons from GAP-EDL-5.

### 2.6. Synthesis of 5-N-[Amino-3-ethoxyestradiol]-Glutamoyl-L-Glutamic acid pentapeptide (GAP-EDL)

**Figure 8:**
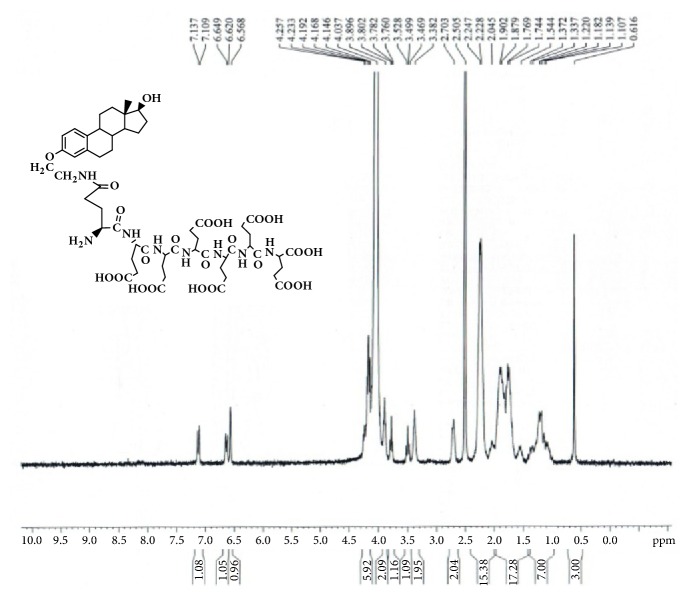
Synthesis of GAP-EDL.

**Figure 9:**
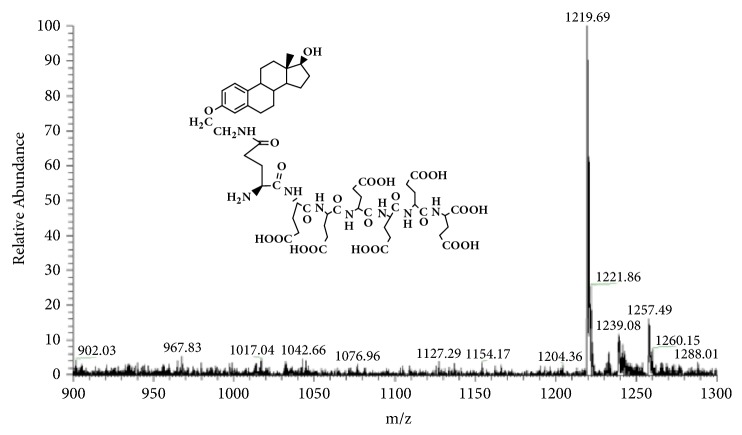
Mass Spectrum of GAP-EDL (C_55_H_78_N_8_O_23_, 1218.5; found [M+H] 1219.7).

**Figure 10:**
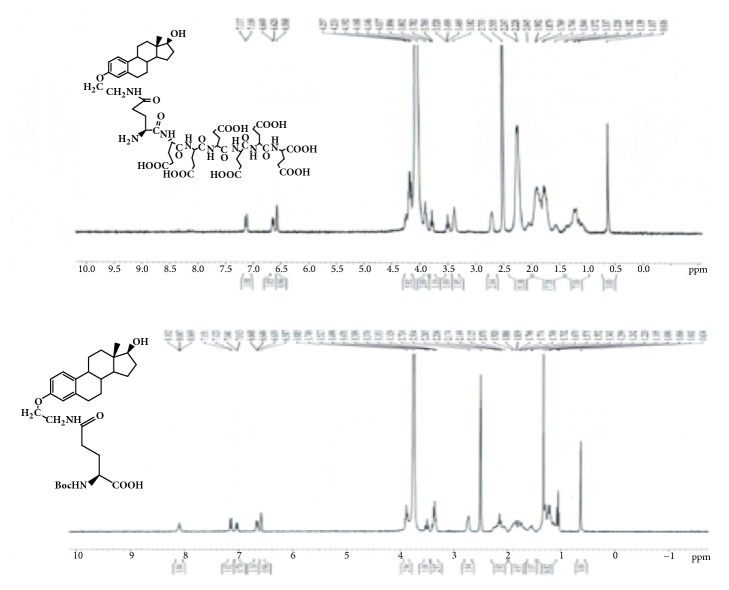
^1^H-NMR of GAP-EDL and GAP-EDL-4.

**Figure 11:**
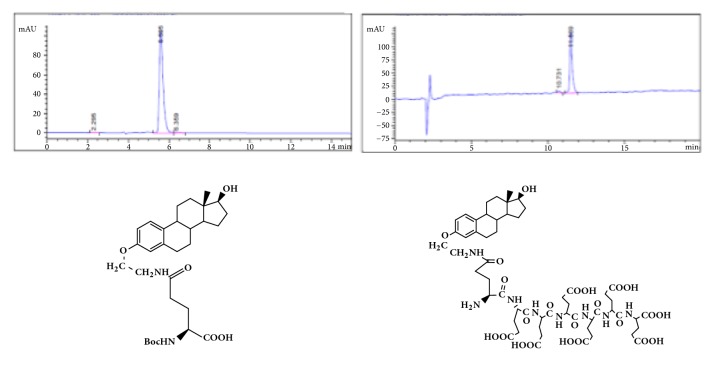
HPLC of GAP-EDL-4 (left) and GAP-EDL (right).

GAP-EDL-5 (0.560g, 0.327 mmol) was dissolved in anhydrous DCM (11mL) at 0-5°C under nitrogen atmosphere. Trifluoroacetic acid (2.8mL, 37.702mmol) was added. The mixture was stirred at room temperature for 16-18h. The solvent was removed under reduced pressure and the residue was washed with diethyl ether to obtain the crude product. The crude product was purified by prep-HPLC using the gradient elution VA:VB=95:5-70:30 (A phase: 0.1% TFA in water solution; B phase: 0.1% TFA in acetonitrile). After lyophilization, GAP-EDL was obtained as white solid (0.214g, 0.176mmol, 53.7%yield). ^1^H-NMR (300MHz, DMSO+D2O): 7.12 (d, J=8.6Hz,1H), 6.64 (d, J=8.6Hz, 1H), 6.57 (s, 1H), 4.10-4.26 (m, 6H), 3.85-3.94 (m, 2H), 3.76-3.80 (m,1H), 3.47-3.53(m, 1H), 3.32-3.41(m, 2H), 2.66-2.76 (m, 2H), 2.00-2.34 (m, 16H), 1.48-1.98 (m, 18H), 1.00-1.40 (m, 7H), and 0.61(s, 3H); LC-MS was calculated for C55H78N8O23, 1218.5, and found [M+H] 1219.7. The proton NMR and mass spectrum are shown in Figures 8 and 9. To ascertain the structure of GAP-EDL, the proton NMR of GAP-EDL-4 (monomer) was compared to GAP-EDL (Figure 10). There were chemical shifts from the aromatic protons at 7ppm seen both in GAP-EDL-4 and GAP-EDL. HPLC analysis showed that there were differences of the absorption (210nm vs 239nm) and retention time between GAP-EDL and GAP-EDL-4 (Figure 11).

### 2.7. Synthesis of ^68^Ga-GAP-EDL

**Figure 12:**
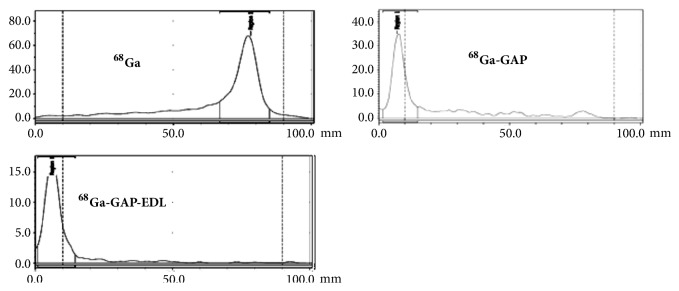
ITLC analysis of ^68^GaCL3, ^68^Ga-GAP and ^68^Ga-GAP-EDL (Polyamide, eluant: saline).

^68^GaCl_3_ was obtained from a ^68^Ge/^68^Ga generator eluted with HCL (ranging from 0.01N-1N). For instance, ^68^GaCl_3_ was eluted from a ^68^Ge/^68^Ga generator (Ithemba, South Aferica) with 0.3N and 0.6NHCL (10mL). On the following day, the elusion volume (0.3N or 0.6NHCL, 6 mL) was distributed in a 12-tube (0.5mL/tube). Each tube was counted for its radioactivity. The highest activities in the fractions between 4 and 6 were combined. In the consecutive cycle, the generator is eluted again with 6mL HCL and collected at these specific fractions. Based upon previous elution profile, an aliquot of ^68^GaCl_3_ (0.5ml in 0.6NHCL, 6.70 mCi) was added to the solution of GAP-EDL (0.1mg) in 0.8 ml NaOAc (2.5M), and pH value was 4-5. The solution was heated at 70°C for 10 min. After cooling, radiochemical yield and purity were determined by ITLC (Polyamide-6-layer sheets, cat. 30149864, Sinopharm Chemical Reagent Co., Shanghai, China), eluted with saline. Radiochemical yield and purity were 100% with Rf value 0.01. Under the same ITLC conditions, the Rf value for ^68^Ga was more than ^68^Ga-GAP and ^68^Ga-GAP-EDL (Figure 12). ^68^Ga chloride eluted from a generator remains at ^68^Ga chloride form under pH 2. ^68^Ga chloride migrates using saline as a mobile phase. However, ^68^Ga chloride forms colloidal when pH changes due to dilution. The colloidal ^68^Ga (Ga_2_O_3_) stays at origin using saline as a mobile phase.

### 2.8. In Vitro Cellular Uptake Assays

In vitro cellular uptake studies of ^68^Ga-GAP-EDL in breast tumor cells were performed. Breast cancer cell lines with high (MCF7; ER+) and low (SK-BR-3; ER-) estrogen receptor densities were used to determine the sensitivity and specificity for the cellular uptake assay. Briefly, for sensitivity assay, breast tumor cells (50,000 cells/well, 12 wells) were added with ^68^GaCl_3_, ^68^Ga-GAP, and ^68^Ga-GAP-EDL (4 uCi/80uL/well, 4ug/well). The cells were incubated for 0.5-2 hrs. To demonstrate that cellular uptake of ^68^Ga-GAP-EDL was via an ER-mediated process (specificity assay), breast tumor cells (50,000 cells/well) were treated with 0, 15, 150, and 300 *μ*mol/L of cold estrone (in DMSO) for 30 minutes followed by adding ^68^Ga-GAP-EDL (4*μ*g/well, 4*μ*Ci/well) and incubated up to 90 minutes. After incubation, the supernatant was collected. The cells were washed twice with ice cold PBS (1 ml) and trypsin EDTA (0.1 ml). After 2 min, the cells were collected. The wells were washed twice with cold PBS (0.5ml). The total volume of cell supernatant and the total volume containing cells were transferred to test tube to count the activity separately. Each piece of data represents an average of three measurements. Percentage of uptake (%)=cell activity/(cell supernatant+ cell activity)*∗*100% was calculated.

### 2.9. Micro-PET/CT Imaging Studies in MCF-7 Breast Tumor-Bearing Mice

To assure stability of ^68^Ga-GAP-EDL, ^68^Ga-GAP-EDL was incubated at 37°C in saline up to 120 min, followed by ITLC analysis. ^68^Ga_2_O_3_ could form colloidal particles during ^68^Ga-GAP-EDL synthesis which decreased tumor uptake, thus, fresh ^68^Ga chloride (pH<2) was used as a control in ITLC system. For in vivo stability, athymic MCF-7 tumor-bearing nude mice (n=2) were administered ^68^Ga-GAP-EDL (0.14mg per 30*μ*Ci, 0.1 mL, iv) and the tissue biodistribution was conducted at 90 min post-administration. The selected tissues were excised, weighed and counted for radioactivity by gamma counter. Each sample was calculated as percentage of the injected dose per gram of tissue wet weight (%ID/g). Counts from a diluted sample of the original inject were used for reference.

For PET imaging studies, athymic nude mice (n=2 per compound) inoculated with MCF-7 cells (s.c. 10^6^ cells/mouse) at the left hind legs. When tumor reached 0.7cm, the tumor-bearing mice were anesthetized with 2% isoflurane on prone position. ^68^Ga chloride (5mCi) was obtained by eluting a ^68^Ge/^68^Ga generator (20mCi size from Eckert Ziegler, Germany) with HCL (0.1N, 1.5 mL). Labeling GAP (1 mg) and GAP-EDL (1mg) was achieved by dissolving both compounds in sodium acetate (0.4 mL, 0.1N, pH 5.4), followed by adding ^68^Ga chloride (1mCi, 0.3mL). Both compounds were sat at 37°C for 20 min. For animal imaging studies, the concentrations were diluted to 1.5mL/mg by adding saline (0.9%). Each mouse was administered with ^68^Ga-GAP and ^68^Ga-GAP-EDL (0.14mg per 100*μ*Ci, 0.2 mL, iv). ^68^Ga-GAP and ^68^Ga-GAP-EDL imaging studies were performed with a Siemens Inveon micro-PET/CT scanner (Siemens Medical Systems, Hoffman Estates, IL). Three serial 30-minute trans-axial PET images were obtained. All corrections for attenuation, scatter, and dead time were applied to generate quantifiable images. Regions of interests were then drawn on the tumor tissue and muscle regions and time activity curves were generated, the counts per pixel were used to determine tumor tissue-to-muscle tissue count density ratios.

## 3. Results

### 3.1. Chemistry

**Scheme 1:**
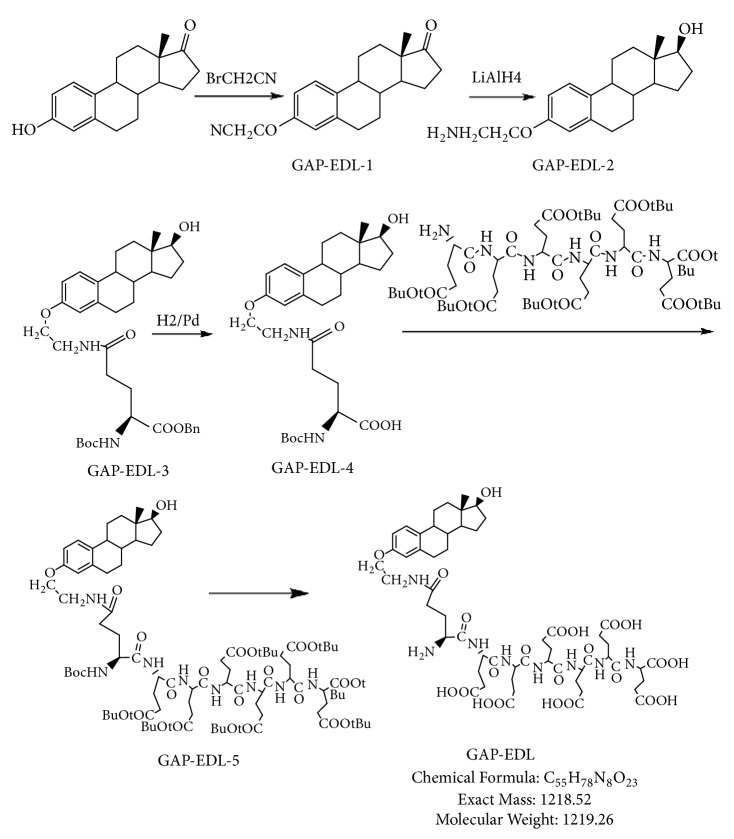
Efficient Synthesis of GAP-EDL.

The synthetic scheme of GAP-EDL is shown in Scheme 1. The analytical data for structural determination of GAP-EDL analogues was shown in Figures 1–12. Aspects of the efficient synthesis provide novel methods for preparing a glutamate-estradiol conjugate and a glutamate peptide-estradiol conjugate. To initiate the efficient synthesis, the position-3 of estrone was reacted with bromoacetonitrile to yield cyano analogue of estrone (GAP-EDL-1). GAP-EDL-1 was then reduced to afford 3-aminoethoxy estradiol (GAP-EDL-2). For preparing a glutamate-estradiol conjugate, the method used an amino estradiol with an amino and 1-carboxylic acid protected glutamate in organic solvent, thereby producing the gamma glutamoyl-estradiol conjugate (GAP-EDL-4). For preparing a glutamate peptide-estradiol conjugate, the method used was by reacting the gamma glutamoyl-estradiol (GAP-EDL-4) with all carboxylic acid protected glutamate pentapeptide comprising coupling agents. Thus, estradiol is positioned at the first glutamate of the glutamate peptide-estradiol in the finished product (GAP-EDL). During the synthesis of GAP-EDL-3, it is unlikely that protected glutamate might be conjugated with 17-OH group of amino-EDL. However, the NMR data showed the conjugation produced an amide bond, not an ester bond. Also, the ester group could not sustain at the hydrogenation process in the next step (GAP-EDL-4). The total synthesis of GAP-EDL was six steps with overall yield 5.7%. The GAP-EDL was analytically pure as proven by NMR (Figure 8), mass spectra (Figure 9), and HPLC (Figure 11).

### 3.2. Cell Uptake Assays

There was a marked increase in the uptake of ^68^Ga-GAP-EDL compared with the uptake of ^68^GaCl_3_ and ^68^Ga -GAP in breast cancer cells (Table 1). The improved synthetic method makes a pure GAP-EDL and has greatly enhanced specificity in cell uptake studies. The uptake of ^68^Ga-GAP-EDL was higher than ^68^Ga-GAP both in ER (+) MCF7 cells and ER (-) SK-BR-3 cells. At 30 min, the uptake of ^68^Ga-GAP-EDL in ER (+) MCF7 cells was significantly higher than ER (-) SK-BR-3 cells but not at 1 and 2hrs. The average cell uptake of ^68^Ga-GAP-EDL of the improved synthesis (Table 1) was at least 5-fold higher than the previous reported synthesis [11]. In specificity assay, the cell uptake of ^68^Ga-GAP-EDL could be blocked by estrone, particularly at 15, 150, 300 *μ*mol/L. Again, MCF-7 has more decreased uptake than ER (-) SKBR-3 particularly at 15*μ*mol/L of estrone (Table 2). The decreased uptake demonstrated the cellular uptake of ^68^Ga-GAP-EDL was via an ER-mediated process.

### 3.3. Micro-PET/CT Imaging and Biodistribution Studies in MCF-7 Breast Tumor-Bearing Mice

**Figure 13:**
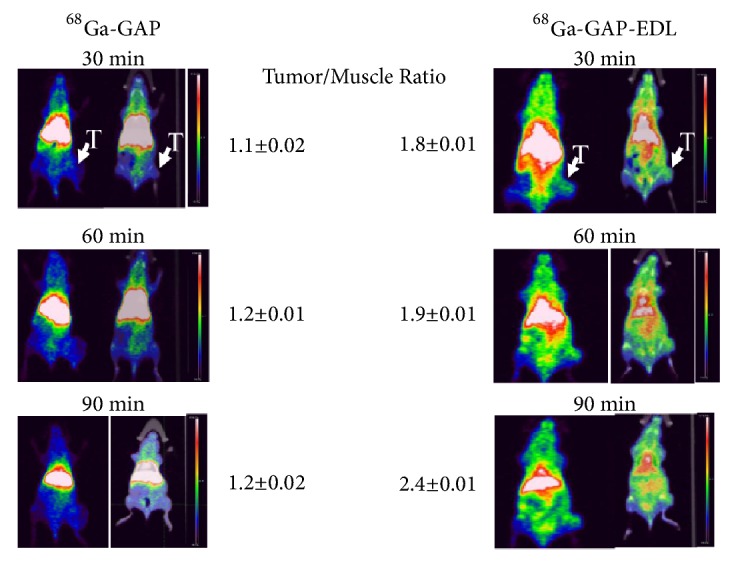
Micro-PET/CT analysis showed that ^68^Ga-GAP-EDL had higher tumor-to-muscle ratios than ^68^Ga-GAP in breast tumor-bearing mice.

MCF 7 cell line was well established for ER (+) cells and was used in cell uptake studies; thus, it was selected for an animal imaging model. In vitro ITLC stability analysis revealed that ^68^Ga-GAP-EDL stayed at origin (Figure 12) whereas free ^68^Ga chloride migrates from 30min to 120 min. PET imaging studies showed that ^68^Ga-GAP had less tumor uptake than ^68^Ga-GAP-EDL at 30-90 min post-administration in breast tumor model by visualization (Figure 13). Preliminary biodistribution of ^68^Ga-GAP-EDL at 90 min revealed the comparable (tumor-to-muscle) data to imaging findings (Table 3).

## 4. Discussion

The discovery of ERs in binding and responding to individual hormonal pathways benefits patients in the treatment of oxidative stress induced ROS, inflammation, and cancer. High ERs were overexpressed in the cytosol during cancerous progression. Glutamate receptor/transporter systems were known to have overexpressions due to high demand of GSH in disease status. Poly-l-glutamic acid (PG) with repeated glutamate units has been used as a tumor-aiming drug carrier for different hydrophobic cancer chemotherapeutic agents [13–21]. PG have other advantages such as excellent water solubility, biocompatibility, nonimmunogenicity, biodegradability to glutamate, and a high drug loading capability due to their multiple carboxyl side groups [22–25]. In order to enhance specificity in targeting ER (+) system, conjugation of EDL to GAP would provide a dual target purpose. First, an enhanced uptake of ^68^Ga-GAP-EDL may occur through cell surface glutamate transporter/receptor system. Secondly, ^68^Ga-GAP-EDL may target ERs faster due to enhanced internalization.

Regarding synthetic production of GAP-EDL for ^68^Ga labeling, when conjugation was produced in aqueous conditions, purification of the GAP-EDL may sometimes present a challenge. Purification in aqueous conditions can be achieved using, for example, size exclusion chromatography, or dialysis with membranes of particular molecular weight cut-offs; for instance, dialysis is generally most effective when separating species of molecular weights of 1000 g/mol or higher. Nevertheless, this method of purification often isolates not only the desired agent but also any other species that may pass through the membrane. Receptor-based imaging agents require high specific activity (>0.1 Ci/umol) in order to overcome nonspecific protein binding. Therefore, introduction of impurities into receptor-based imaging agents may be problematic in their clinic uses. For instance, if an imaging agent incorporating a radionuclide is thought to be pure but actually contains impurities that also incorporate a radionuclide, the proper measurement or detection of the imaging agent may be obscured or rendered false due to the presence of the impurity. This is particularly true when using polypeptide such as polyglutamate as a drug carrier. Commercially available polyglutamate has a wide range molecular weight which contains a mixture of various polyglutamate. This makes difficulty in the structure determination of the EDL position in the molecule. In addition, the conjugation reaction is frequently done in an aqueous condition in which the product purity is not optimized.

For preparing a glutamate peptide-estradiol conjugate, the protected carboxylic acid of glutamate-estradiol conjugate is selectively deprotected and reacted with all acid protected glutamate pentapeptide ester using coupling chemicals such as DIPEA and BOP. These coupling agents are able to conjugate estradiol to the glutamate peptide at the first glutamate using the synthetic route described in Scheme 1. In some aspects, these synthetic methods may obviate the need of adding protecting groups to glutamate and glutamate pentapeptide and increase process efficiency and purify of the final product as compared to other methods as described in US Patent application 20060246005, WO 2006107794 A2 [10–12]. To generate a metal ion labeled-glutamate peptide-estradiol conjugate, the metal ion selected was a gallium ion, a radionuclide for PET imaging. The site to be imaged may be a tumor or endometriosis or an ER-enriched tissue such as ovaries and uterine tissue. ER (+) cancers are breast cancer, lung cancer, prostate cancer, ovarian cancer, uterine cancer, cervical cancer, and endometrial cancer. To demonstrate that GAP-EDL is suitable for commercialized generators, we have used two different generators to label GAP-EDL. Both generators were able to synthesize ^68^Ga-GAP-EDL. ^68^Ga-GAP-EDL was stable in saline at least up to 120 min. Our in vitro cellular uptake assays demonstrated that the uptake of ^68^Ga-GAP-EDL was higher than ^68^Ga-GAP both in ER (+) MCF7 cells and ER (-) SK-BR-3 cells. MCF-7 showed early binding that is retained, whereas SKBR-3 shows higher binding as a function of time. However, the increased uptake of ^68^Ga-GAP-EDL in ER (+) MCF7 cells could be significantly blocked by estrone at 15*μ*mol/L, but not ^68^Ga-GAP, indicating the uptake mechanism was via an ER-mediated process. In vivo imaging findings indicated that ^68^Ga-GAP-EDL could image ER (+) tumors. Further studies warrant evaluation of whether ^68^Ga-GAP-EDL remains intact and associates with ER once internalized.

In summary, the efficient synthesis of GAP-EDL conjugate for molecular imaging of ER (+) disease was achieved. GAP-EDL integrating diagnostic imaging instrument helps to understand the dynamic changes in ER (+) pathway-activated systems leading to tissue degeneration, inflammatory, and proliferative disorders and to improve patient diagnosis, therapy, and prognosis. The efficient synthetic methods can be further prepared in pharmaceutical formulations and kits using chemical procedures. This precision imaging agent allows delivering personalized therapeutics on the basis of individual genetic make-up, biochemistry, and molecular blueprint associated to each patient's disease.

## Figures and Tables

**Table 1:** In vitro cell uptake assays (an average of three measurements).

	**MCF-7**			**SK-BR-3**		
	^68^GaCl_3_	^68^Ga-GAP-EDL	^68^Ga-GAP	^68^GaCl_3_	^68^Ga-GAP-EDL	^68^Ga-GAP
**30min**	1.35±0.35	12.91±0.4*∗*	2.20±0.73	1.74±0.21	3.63±1.02	1.84±0.27
**1h**	1.79±0.28	10.61±2.34	3.20±1.17	1.80±0.20	9.28±0.49	3.11±1.29
**2h**	1.63±0.33	10.17±1.38	2.43±0.71	2.21±0.11	10.65±0.78	2.45±0.21

*∗* Significant difference between corresponding groups (t-test, P<0.05).

**Table 2:** In vitro cell blocking assays with 68Ga-GAP-EDL (an average of three measurements).

**Estrone (umol/L)**	**MCF-7**	**SK-BR-3**
**0**	14.78±3.10	9.38±1.95
**15**	7.96±2.09	8.55±3.55
**150**	2.95±0.56	2.68±1.00
**300**	2.95±0.43	2.84±1.00

**Table 3:** Biodistribution of 68Ga-GAP-EDL at 90 min in MCF-7 tumor-bearing mice (n=2).

**Tissue**	**Mouse 1 (**%**ID/g)**	**Mouse 2 (**%**ID/g)**	**Mean ± SD (**%**ID/g)**
Uterus	9.08	9.85	9.47 ±0.55
Ovary	10.26	8.53	9.39 ±1.23
Kidney	7.55	7.60	7.58 ±0.04
Tumor	6.42	8.42	7.42 ±1.41
Bone	6.94	7.33	7.14 ±0.28
Liver	6.57	7.22	6.89 ±0.45
Lung	7.59	6.13	6.86± 1.03
Heart	6.01	6.27	6.14 ±0.18
Spleen	4.56	5.47	5.02± 0.64
Muscle	3.20	2.95	3.07 ±0.18
Blood	2.32	2.33	2.33 ±0.01
Brain	0.63	0.75	0.69 ±0.08

## Data Availability

The data used to support the findings of this study are available from the corresponding author upon request.
